# Adaptations to mentoring and peer mentor training at the medical faculty during the COVID-19 pandemic

**DOI:** 10.3205/zma001404

**Published:** 2021-01-28

**Authors:** J. Zibold, J. A. Gernert, L. J. U. Reik, L. M. Keidel, T. Graupe, K. Dimitriadis

**Affiliations:** 1 University Hospital LMU Munich, Institute for Medical Education, Munich, Germany; 2 University Hospital LMU Munich, Department of Neurology, Munich, Germany; 3 University Hospital LMU Munich, Institute for Stroke and Dementia Research (ISD), Munich, Germany

**Keywords:** COVID-19, corona virus, mentoring, mentors, medical education, online education

## Abstract

**Objectives: **The COVID-19 pandemic has led to major adjustments in health care systems and significantly affected medical education. Accordingly, our mentoring program *MeCuM-Mentor* had to expand its virtual elements, in order to continue to meet the needs for mentoring at the medical faculty of the *Ludwig-Maximilians-University Munich*.

**Methods: **Here we report on our recently implemented online formats to facilitate training for currently coached peer mentors, as well as the introduction of an online consultation hour and a new social mentoring event called *PubQuiz*.

**Results: **First results demonstrated feasibility of the above-mentioned virtual formats, which were positively rated by the participants in small voluntary evaluation questionnaires. Utilization rates indicate existing need for mentoring during the pandemic. In addition, the new event *PubQuiz *promotes social interaction among peers during isolation due to COVID-19.

**Conclusion: **With the transition to online formats, mentoring at the Medical Faculty could be continued during COVID-19. The newly introduced mentoring event *PubQuiz *will be repeated. However, it remains unclear to what extent online formats can replace in-person one-to-one mentoring conversations or peer mentoring meetings.

## Zusammenfassung

**Zielsetzungen: **Die COVID-19-Pandemie hat zu großen Anpassungen in den Gesundheitssystemen geführt und die medizinische Ausbildung erheblich beeinträchtigt. Dementsprechend musste unser Mentoring-Programm MeCuM-Mentor seine virtuellen Elemente erweitern, um dem Mentoring-Bedarf an der Medizinischen Fakultät der *Ludwig-Maximilians-Universität München* weiterhin gerecht zu werden.

**Methoden: **Hier berichten wir über unsere neu implementierten Online-Formate zur Erleichterung der Ausbildung studentischer Peer-Mentor*innen sowie über die Einführung einer Online-Sprechstunde und einer neuen sozialen Mentoring-Veranstaltung namens *PubQuiz*.

**Ergebnisse: **Erste Ergebnisse zeigen die Realisierbarkeit der virtuellen Formate, die von den Teilnehmern positiv bewertet wurden. Die Auslastung der Formate deutet auf einen bestehenden Bedarf an Mentoring während der Pandemie hin. Darüber hinaus fördert die neue Veranstaltung *PubQuiz *die soziale Interaktion unter den Studierenden während der Kontaktbeschränkungen aufgrund von COVID-19.

**Schlussfolgerung: **Mit dem Übergang zu Online-Formaten konnte das Mentoring an der Medizinischen Fakultät während COVID-19 fortgesetzt werden. Das neu eingeführte *PubQuiz *wird wiederholt. Es bleibt jedoch unklar, inwieweit Online-Formate persönliche Mentoring-Gespräche oder Peer-Mentoring-Treffen ersetzen können.

## Introduction

Since March 2020 any large in-person class or university social event was prohibited due to COVID-19 pandemic. Transition into online curricula, lack of practical experience and limitations in peer exchange, led to uncertainties and insecurities among medical students. The value of mentoring in medical education has been widely acknowledged [1]. First publications indicated an increased demand for mentoring during the pandemic [2], [3]. Furthermore, it has been emphasized that new formats, adjusted to the restrictions of a pandemic, are necessary [4]. Thus, our well-established mentoring program *MeCuM-Mentor *[5] adapted its virtual elements in order to meet need for mentoring. We here present new implemented online formats for peer mentor training, online mentoring consultation and a social mentoring event which all aim to support medical students during COVID-19.

## Project description

*MeCuM-Mentor* is the mentoring program for over 4000 students at the medical faculty of the *Ludwig-Maximilians-University Munich* [6]. It is based on two main pillars: a one-to-one mentoring program comprising a large number of mentoring relationships (757 matches physicians – senior students; 365 matches student peer mentors – junior students) and well-established peer mentoring events, covering various topics (such as career planning, study options) organized by students [7], [8], [9]. While the one-to-one matchmaking process was already based on an online tool [10], all mentoring events and training of new peer mentors used to take place face-to-face. Adapting to the pandemic our mentoring program expanded its online elements to provide a nearly in-person mentoring interaction via the platform Zoom:

1. First important step was to convert the pre-planned training for the currently coached peer mentors into a monthly virtual course via Zoom. Subsequently, they could accompany digital implementation of mentoring events. An online evaluation questionnaire was sent by e-mail to all currently trained peer mentors at the end of the semester.
2. Due to an above-average number of inquiries from students via e-mail to the coordinators of the mentoring program, a weekly consultation hour via Zoom was initiated in a second step. There, students could regularly and easily address their questions in individual one-on-one talks with a coordinator of the mentoring program.
3. Furthermore, a new event called *PubQuiz* with regard to the psychosocial consequences of COVID-19 was initiated. As already pointed out by *Gotian *2020: “Most importantly, remember the human and empathetic part of the mentoring relationship” [2]. In seven teams of five participants, consisting of physician mentors, peer mentors and students, quiz questions were answered in break out rooms via Zoom. The aim of the event was to create an evening program despite the social isolation caused by COVID-19, which, besides the fun factor of the quiz, should promote the exchange between mentors and mentees and attract new students to the mentoring program. An online evaluation was conducted using the survey tool from Zoom.

## Results

Utilization rate of all online implemented elements showed existing demand for mentoring among students. In addition, feedback was positive:

1. In the virtual peer mentoring course, topics were: adaptation by COVID-19, evaluation of mentoring relationships, planning of the upcoming semester as well as discussions on new projects. In a small voluntary evaluation questionnaire among the currently trained peer mentors (n=12 of 35 peer mentors, return rate 34.3%), the majority pleaded for future continuation of the meetings with the peer mentor group not only in person (75%) but also via Zoom (66.7%). In the context of their one-to-one peer mentoring, student mentors stated that they continued meetings virtually (including telephone calls (25%), video calls e.g. via Zoom (50%), text message (25%)).
2. The weekly consultation hour via Zoom was regularly used by students. Topics included advice on mentoring and matching, information on how to apply as a peer mentor, and questions about different projects.
3. The *PubQuiz *was evaluated with a small questionnaire (n=21 of 35 participants, response rate 60%), in which 85.7% rated the event as excellent. The vast majority also pleaded for repetition – both in virtual form (85.7%) and face-to-face (60%). Nine participants (42.9%) also indicated that this encouraged them to find their own mentor.

## Discussion

The reorganization of our mentoring program by means of online platforms made it possible to keep mentoring running and to stay connected with all our stakeholders despite COVID-19. Moreover, training of peer mentors was warranted, which helped maintaining established projects and organizing new ones. Online formats offer the option to plan mentoring conversations more flexible and thus exchange experiences more often. However, in such virtual meetings the impact of an in-person contact is missing. Evaluation results of *PubQuiz *showed that physician mentors and students enjoy socializing even in a virtual casual atmosphere. In future, such event could be both offered virtually and in-person, in order to accommodate all preferences and foster a more informal exchange between mentors and mentees.

## Conclusion

A transition into online mentoring was successfully demonstrated and positively evaluated by participants. Both new mentoring consultation and training of the peer mentors could be smoothly implemented online. With *PubQuiz*, a new event was created that allows mentoring conversations in a novel context, focusing on the dimension of social interaction. Thus, we provide students with a valuable reference point even during COVID-19. Due to the continuing interest in the *PubQuiz*, a repetition is already planned. To what extent virtual meetings can achieve similar outcomes and therefore replace face-to-face events in the mentoring context has to be investigated in future prospective randomized trials.

## Competing interests

The authors declare that they have no competing interests. 
